# Supplementary material for: Genomic insights into the comorbidity between type 2 diabetes and schizophrenia
Source: Schizophrenia (Heidelb). 2024 Feb 21;10(1):22. doi: 10.1038/s41537-024-00445-5 (PMC10881980; doi:10.1038/s41537-024-00445-5)
Supplement: Supplementary file 1 — Supplemental Material File #1 [file 41537_2024_445_MOESM1_ESM.docx]

# Supplemental tables

**Table S1**: Genetic correlation analysis between type 2 diabetes (T2D) and schizophrenia (SCZ) using LD score regression.

**Table S2A:** Mendelian randomization analysis between type 2 diabetes (T2D), schizophrenia (SCZ) and endophenotypes of T2D using inverse variance weighted, weighted median and MR-Egger.

**Table S2B:** Steiger-filtered results of Mendelian randomization analysis between type 2 diabetes (T2D), schizophrenia (SCZ) and endophenotypes of T2D using inverse variance weighted.

**Table S2C:** European ancestry only results of Mendelian randomization analysis between type 2 diabetes (T2D), schizophrenia (SCZ) and endophenotypes of T2D using inverse variance weighted.

**Table S3A:** Univariate Mendelian randomization analysis between childhood and adulthood body mass index (BMI) and type 2 diabetes (T2D) or schizophrenia (SCZ) using inverse variance weighted, weighted median and MR-Egger.

**Table S3B:** European ancestry only univariate Mendelian randomization analysis between childhood and adulthood body mass index (BMI) and type 2 diabetes (T2D) or schizophrenia (SCZ) using inverse variance weighted, weighted median and MR-Egger.

**Table S3C:** Steiger-filtered univariate Mendelian randomization analysis between childhood and adulthood body mass index (BMI) and type 2 diabetes (T2D) or schizophrenia (SCZ) using inverse variance weighted.

**Table S4A:** Multivariate Mendelian randomization analysis between childhood and adulthood body mass index (BMI) and type 2 diabetes (T2D) or schizophrenia (SCZ).

**Table S4B:** European ancestry only multivariate Mendelian randomization analysis of causal inference analysis between childhood and adulthood body mass index (BMI) and type 2 diabetes (T2D) or schizophrenia (SCZ).

**Table S5**: Overview of genomic loci that colocalize between type 2 diabetes and schizophrenia with a posterior probability (PP4) > 0.8.

**Table S6A**: Scoring of all genes within genomic loci that colocalize between type 2 diabetes (T2D) and schizophrenia (SCZ).

**Table S6B**: Scoring of genes showing involvement in both type 2 diabetes (T2D) and schizophrenia (SCZ) with total score of at least 3.

**Table S6C**: Scoring of putative effector genes showing involvement in both type 2 diabetes (T2D) and schizophrenia (SCZ) with total score of at least 4.

**Table S7A:** Mendelian randomization analysis between expression of putative effector genes in disease-relevant tissues and type 2 diabetes (T2D) or schizophrenia (SCZ).

**Table S7B:** European ancestry only Mendelian randomization analysis between expression of putative effector genes in disease-relevant tissues and type 2 diabetes (T2D) or schizophrenia (SCZ).

**Table S8**: Gene set enrichment analysis on the genes showing evidence of involvement in both type 2 diabetes and schizophrenia using the Gene Ontology human networks via ConsensusPathDB.

**Table S9A**: List of knockout (KO) mice phenotypes and OMIM terms related to type 2 diabetes.

**Table S9B**: List of knockout (KO) mice phenotypes and OMIM terms related to neuropsychiatric traits.
